# SMART – Sunflower Mutant population And Reverse genetic Tool for crop improvement

**DOI:** 10.1186/1471-2229-13-38

**Published:** 2013-03-05

**Authors:** Anish PK Kumar, Adnane Boualem, Anjanabha Bhattacharya, Seema Parikh, Nirali Desai, Andres Zambelli, Alberto Leon, Manash Chatterjee, Abdelhafid Bendahmane

**Affiliations:** 1 Bench Bio Pvt Ltd., c/o Jai Research Foundation, Vapi, Gujarat, 396195, India; 2 INRA, UMR1165 Unité de Recherche en Génomique Végétale URGV, Evry, F-91057, France; 3 UEVE, UMR Unité de Recherche en Génomique Végétale URGV, Evry, F-91057, France; 4 CNRS, ERL8196 UMR Unité de Recherche en Génomique Végétale URGV, Evry, F-91057, France; 5 Biotechnology Research Centre, Nutrisun Business Unit- Advanta Semillas SAIC, Belcarce, Argentina; 6 National University of Ireland Galway (NUIG), Galway, Ireland

**Keywords:** Sunflower, TILLING, Functional genomics, Oil quality, Fatty acids, Crop improvement

## Abstract

**Background:**

Sunflower (*Helianthus annuus* L.) is an important oilseed crop grown widely in various areas of the world. Classical genetic studies have been extensively undertaken for the improvement of this particular oilseed crop. Pertaining to this endeavor, we developed a “chemically induced mutated genetic resource for detecting SNP by TILLING” in sunflower to create new traits.

**Results:**

To optimize the EMS mutagenesis, we first conducted a “kill curve” analysis with a range of EMS dose from 0.5% to 3%. Based on the observed germination rate, a 50% survival rate *i.e.* LD_50_, treatment with 0.6% EMS for 8 hours was chosen to generate 5,000 M2 populations, out of which, 4,763 M3 plants with fertile seed set. Phenotypic characterization of the 5,000 M2 mutagenised lines were undertaken to assess the mutagenesis quality and to identify traits of interest. In the M2 population, about 1.1% of the plants showed phenotypic variations. The sunflower TILLING platform was setup using Endo-1-nuclease as mismatch detection system coupled with an eight fold DNA pooling strategy. As proof-of-concept, we screened the M2 population for induced mutations in two genes related to fatty acid biosynthesis, *FatA* an acyl-ACP thioesterase and *SAD* the stearoyl-ACP desaturase and identified a total of 26 mutations.

**Conclusion:**

Based on the TILLING of *FatA* and *SAD* genes, we calculated the overall mutation rate to one mutation every 480 kb, similar to other report for this crop so far. As sunflower is a plant model for seed oil biosynthesis, we anticipate that the developed genetic resource will be a useful tool to identify novel traits for sunflower crop improvement.

## Background

Sunflower (*Helianthus annuus* L.), 2n =34, belongs to family *Asteraceae*, with an estimated genome size of 3000 Mbp, and is the fourth most important oilseed crop [1]. It is a native of North America [2] and widely cultivated in the world with an annual production of about 24 million tonnes (http://www.fao.org). The genome size of this crop is large compared to model species like Arabidopsis (125 Mbp), Rice (430 Mbp), Sorghum (750 Mbp), Soybean (1100 Mbp) or Tomato (950 Mbp) (reviewed by [3]). The major drivers of this large genome size are mainly the recent polyploidization event and the important amplification of transposable elements [2,4]. Many wild sunflower species are increasingly used for improvement of cultivated varieties by conventional breeding [5]. With continued biased selection of the cultivated sunflower for traits such as yield, many alleles conferring useful traits got lost. Therefore a method to create variability for breeding this crop, in this era of increasing global food crisis and changing climatic regimes, is highly desirable. For many decades, plant breeders have concentrated their efforts on improvement of sunflower through traditional breeding and recently, molecular mapping has been successfully undertaken for marker assisted breeding (MAS) [6]. Furthermore, chemical mutagenesis has been used by breeders to create variability in crops including sunflower but these have been mostly restricted to dominant traits. Therefore, many desirable mutations that are recessive have been missed during selection [7]. Finally, traits such as oil quality cannot be selected by visual inspection of plants.

Sunflower is one of the major oil seed crops grown all over the world and considerable research efforts have been put to understand lipids biosynthesis and modification. This research mainly focused on (i) the production of more stable sunflower oils for biolubrication (high oleic acid content) and (ii) the increase of healthy substitutes for food industry [8]. The *de novo* synthesis of fatty acids in plant storage tissues is an intraplastidial process in which the multienzyme fatty acid synthase (FAS) complex catalyses a series of enzymatic reactions. In sunflower, the main products of intraplastidial fatty acid synthesis are, first, palmitoyl-ACP (16:0-ACP), which is further elongated by the FAS II complex to produce stearoyl-ACP (18:0-ACP). In turn, this molecule is the substrate for stearoyl-ACP desaturase (SAD) that introduces a double bond in the carbon chain to produce oleoyl-ACP (18:1-ACP). All of these products can be exported from the plastid after the hydrolysis between the acyl molecule and ACP by the acyl-ACP thioesterase. Two types of acyl-ACPs thioesterases have been identified in higher plants such as sunflowers: FatA and FatB. FatA thioesterases preferentially act on long chain fatty acids, and have particularly high specificity for 18:1-ACP and a lower affinity for 16:0-ACP and 18:0-ACP [9]. To address the needs of the confectionery industry for saturated fatty acids, high stearic acid content oils have been developed mainly by genetic modification of the FatA stearoyl-ACP thioesterase and the SAD stearoyl-ACP desaturase [10,11]. Stearic fatty acid is considered cardiovascular neutral and do not modify the plasmatic cholesterol levels in humans [12].

TILLING (Targeting Induced Local Lesion IN Genome) is a technology to detect induced and natural polymorphisms (SNP) in plants [13]. The critical steps in TILLING procedure include the purity of seed material, the quality of the mutagenesis and the size of the mutant collection. TILLING relies on the ability of a group of enzymes, the endonucleases, to detect mismatches in a specific gene ([14]). With the introduction of high throughput TILLING technology ([15]), detection of even rare recessive mutation is possible [16,17]. The significant advantages of TILLING method in creating and discovering new traits is that it significantly costs less for product development compared to transgenic crop plants. This makes it an attractive tool for crop improvement. As sequencing technology is getting advanced, deciphering small changes, like SNPs, which plays an important role in adaptive response and evolution of species is possible [3].

Sunflower, cultivated as a source of vegetable oil and protein is an attractive model for investigating seed oil quality. To investigate such trait, we have developed a reference EMS mutant population under controlled conditions and established a TILLING platform. As proof-of-concept, we screened for mutations in two genes, *FatA* and *SAD*, involved in accumulation of short to medium chain fatty acids and identified 26 induced mutations.

## Results

### EMS mutagenesis

A relatively large TILLING resource comprising 5,000 M2 families was developed for sunflower crop improvement using variety BBS-1. To find the optimum dose of EMS that produce a maximum mutation density without causing extensive sterility and lethality; a ‘kill curve’ analysis was conducted on batches of 100 seeds treated with different EMS doses, form 0% to 3%, during 8 hours (Table 1). At 0.5% and 0.6% EMS, seed germination rate was 54% and 50%, respectively. Drastic plant lethality was observed for seeds treated with an EMS dose greater than 1% and total lethality was observed at 3% EMS. Finally, 0.6% EMS treatment during 8 hours, corresponding to the LD_50_ was retained and tested on large batches of seeds.

**Table 1:** Impact of EMS concentration on seed germination

**EMS dose (%)**	**Treated seeds**	**Seed germination (%)**
0	100	78
0,5	100	54
0,6	100	50
0,75	100	44
1	100	38
2	100	3
3	100	0

A total of 25,000 seeds were mutagenised and sown in pots to raise the nursery for creating the TILLING sunflower genetic resource. About 12,500 M1 plants were obtained, showing 50% lethality at seedling stage. After transplanting, further mortality occurred and a total of 6,500 M1 plants produced seeds, among which 5,000 M1 plants produced a good seed set. M2 seeds were harvested from the individual M1 plants. In total, 5,000 M2 seed stocks were harvested and corresponding M3 seeds were produced.

### M2 plant phenotyping

**Figure 1:**
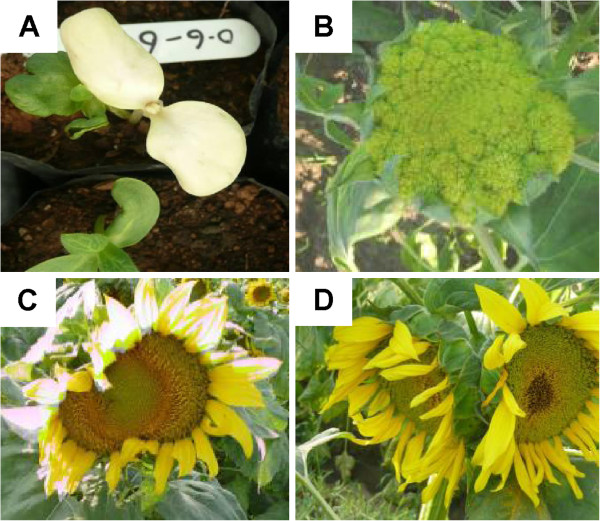
**Phenotypes identified in SMART genetic resource. A**). albino seedling, **B**) sterile flower head, **C**) fused flowers and **D**) twin flowers.

Ten seeds per M2 family were sown in pots to raise the nursery and to screen for visual developmental phenotypes. After 4 weeks, plants were transferred to the fields and about 95% of the M2 plants were recovered. The phenotypic scoring was based on the observation of each plant, using the untreated sunflower phenotype as a reference. The phenotypic screen included dwarfism, plant height, flowering pattern, the leaf architecture and sterility. The data collected are shown in Table 2. Photographs were taken at regular intervals for record purposes (Figure 1). A range of phenotype was observed and some are described below. Compared to the untreated sunflower, 10 and 4 plants showed dwarf and tall phenotype, respectively (Table 2). Some of these dwarf plants can be used in creating hybrids with enhanced yield and dwarf stature. For the flower phenotype, we identified 12 plants with large flower heads (potential to increase yield), 13 plants with twin flowers, 8 plants with fused and/or closed flower and 3 plants with upright flower (Table 2 and Figure 1B-D). Three plants harbored variation in flower color, 1 plant produced small seeds and 3 plants were sterile as both disc and ray florets were missing. In total 57 M2 plants, representing 1.1% of the population, showed at least one altered trait.

**Table 2:** List of mutant phenotype classes

**Category**	**Sub-category**	**Nb of plants**	**%**
Plant height	Tall	4	0,08
	Dwarf	10	0,2
Flowering	Large flower head	12	0,24
	Twin flower	13	0,26
	Fused flower	2	0,04
	Closed flower	6	0,12
	Sterile Flower	3	0,06
	Colour	3	0,06
	Upright flower	3	0,06
Leaf architecture	Aberrant	1	0,02
Total		57	1,1

### Mutation discovery by TILLING

Screening for mutations in the two genes, *FatA* and *SAD*, was used as proof-of-concept to determine the quality of the sunflower mutant collection and to estimate the mutation density. Both of these genes control production of short to medium chain fatty acids [18,19]. SAD (Stearoyl-ACP desaturase) controls the production of stearic acid over oleic acid and inhibition of this enzyme leads to an increase stearic acid synthesis and a reduction of oleic acid production [18]. There are two isoforms of acyl-ACP thioesterase, FatA and FatB which dictate the length of medium chain fatty acids production. FatA controlling essential role in chain termination of fatty acid synthesized [19] was used in the present study.

**Figure 2:**
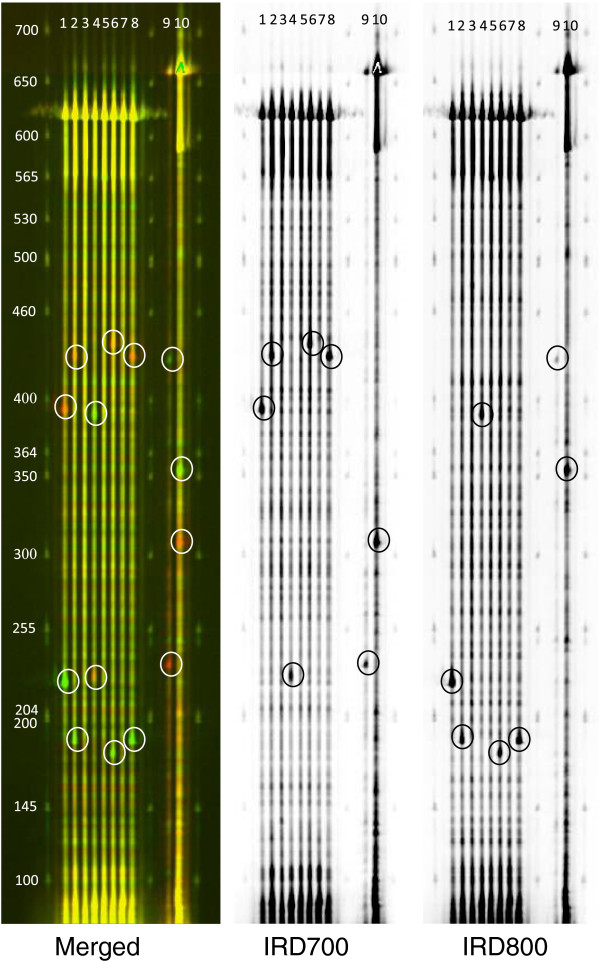
Li-CoR Gel electrophoresis and identification of mutants.

**Figure 3:**
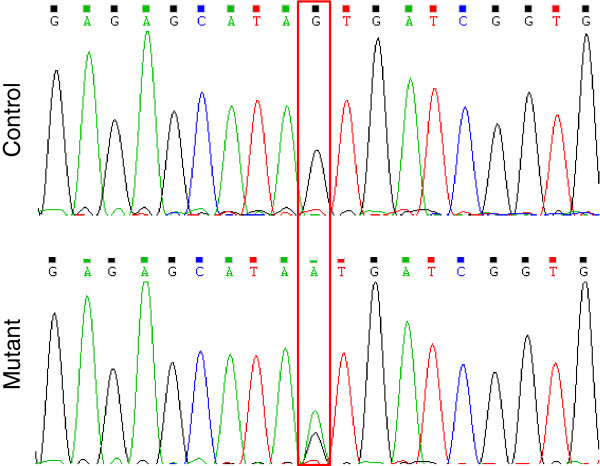
**Mutant detection by sequencing.** Individual plants from the same M2 family have been sequenced to indentify the nucleotide substitution. Sequences of a control plant (top) and a heterozygous mutant (bottom) are shown.

We screened for mutations in the whole exonic and promoter regions of *FatA* and *SAD* genes. Mutations were detected in the amplified targets using the mismatch-specific endonuclease ENDO1 as previously described (Figure 2, [20,21]). TILLING of *FatA* yielded 12 independent point mutations, which correspond to 6 silent, 3 missense, 1 stop codon and 2 promoter region mutations (Table 3). TILLING of *SAD* yielded 14 independent point mutations, which correspond to 8 silent and 6 missense mutations (Table 3). All the identified mutations were confirmed by sequencing (Figure 3) and, as expected for EMS mutagenesis, single nucleotide substitutions were identified both in coding and non-coding regions and classical EMS mutations G to A and C to T were induced (data not shown).

**Table 3:** **Types of induced mutation in*FatA*and*SAD*and mutation frequency**

**Target genes**	**Function**	**Fragment Size (bp)**	**Identified mutations**						**Mutation frequency (1/kb)**
			**Missense**	**Stop codon**	**Silent**	**Intronic**	**Promoter region**	**Total**	
*FatA*	Fatty acid biosynthesis	1,881	3	1	6	1	2	12	1/783
*SAD*	Fatty acid biosynthesis	619	6	0	8	0	0	14	1/221
Total		2,500	9	1	13	1	2	26	1/480

The mutation frequency for each amplicon is calculated as follows:

[(size of the amplicon) × (total number of samples screened)] / (total number of identified mutants). The average mutation frequency was estimated to be one mutation per 480 kb.

We calculated the mutation frequency in the *FatA* and *SAD* genes (Table 3) according to Dalmais *et al.*[20]. We estimated the SMART average mutation density to one mutation every 480 kb. This mutation rate is similar to those reported in the other diploid crops (reviewed by [3]).

The large number of mutations identified in the TILLING screens, *i.e.* 26 independent mutations, confirms that the genetic resource developed is of high quality. Background mutations in the interesting mutant line can be removed by backcrossing with the parental non-mutated line and cleaned lines can be released after trials as a variety. As the mutation is known, the released varieties can be followed in the field by Marker Assisted Selection (MAS). Mutations resulting in STOP codon and in splicing junction of exon-intron boundary are of great potential as found in the present study. Currently bioinformatic tools are available to predict the severity of the non-synomonous mutations [22]. Synonymous substitutions have the potential to alter the production and function of a protein through folding changes, although the exact mechanism and frequency of such event is still a mystery [23].

## Discussion

As the world population continues to grow at a rapid pace, crop varieties with novel traits such as improved growth and yield potential are needed to feed in the future. Plant breeding is an old science which played a significant role during green revolution for its contribution in increasing crop yields. At present, there is skepticism against growing transgenic crops and transgenic technology, mainly because of the high cost incurred in creating a variety due to lengthy regulatory hurdles, which is almost absent with mutation breeding. TILLING provides a way to overcome problems associated with other techniques used in plant improvement like RNAi, over expression of genes, antisense technique, site directed mutagenesis by zinc fingers, T-DNA knockouts and transposon tagging [3,24].

Many useful alleles involved in qualitative and quantitative parameters that exist in natural crop populations have been eliminated in the numerous rounds of selection for targeted traits. Therefore, creation of novel genetic resource by mutagenesis is a useful tool in crop improvement. In this study, we present a sunflower TILLING genetic resource produced by EMS chemical mutagenesis. To set up the sunflower TILLING platform, we developed an EMS mutant collection under controlled condition. The 5,000 M2 mutant collection was phenotyped and genomic DNA was prepared from the mutant plants and organized in pools for bulked screens. The mutant collection phenotyping showed that 1.1% of the mutant plant present at least on altered trait and confirm the quality of the mutagenesis. The sunflower mutant collection could be used for both forward and reverse genetics.

Sunflower has not been sequenced until now, owing to its large genome size (about 3,000Mbp). However, 443,858 ESTs are available at NCBI as of today. Studies by Chapman *et al*. [25] conducted genome scan in cultivated sunflower to predict genes involved in evolution of modern day cultivated variety. Lai *et al*. [26] published SNPs identified from submitted EST information in gene banks. Therefore, comparative genomic analysis can successfully be performed to pinpoint key gene information relating to various traits in this species by comparing known genetic information from other sequenced species.

To validate the quality of mutagenised population, we screened for mutants in *FatA* and *SAD* genes involved in oil biosynthesis. Many other published reports are available today pertaining to changes in seed oil composition in crop varieties. For example, mutations induced in the gene encoding Δ12 fatty acid desaturase (*FAD2*). Screening for *FAD2* variants which increase oleic acid has been confirmed in soybean [27] and novel *FAD2* alleles have been pursued in peanut [28] and sunflower [24]. In the SMART mutant population, the mutation frequency was estimated to be 1/480 kb similar to the recently published reports in sunflower (1/475, [24]) and other crops (Arabidopsis 1/300 kb, Soybean 1/250 kb, Rice 1/265 kb, Tomato 1/322 kb; reviewed by [3]).

Chemical mutagenesis and TILLING approach together can be used to develop plants not only for the purpose of increasing productivity and quality parameters, but also in phytoremediation [29]. TILLING has been successfully used in many crops to obtain useful alleles such as long shelf-life in melon and tomato [13,30-33], improved starch quality in potato [34], virus resistance in tomato and pepper [35-37]. A number of crops are being sequenced at present, sequence information of more than 30 crops are available now and more than hundred crop plants are being sequenced (NCBI Genome Project database; http://www.ncbi.nlm.nih.gov/genomes/leuks.cgi). Many key value-added traits can be obtained in sunflower with candidate gene polymorphism.

## Conclusion

A TILLING genomic resource was developed for cultivated sunflower. Novel mutants having useful mutation in genes can be found by screening the population through high throughput ADP (Allele Discovery Platform) technique as mentioned in this article. Results obtained by screening two proof-of-concept genes are highly promising and confirms that the population is well mutagenized. Additional candidate genes can be screened from the resource developed. Such approach can also be used to develop markers for MAS (Marker assisted selection) strategy in sunflower breeding programs.

## Methods

### Standardization of EMS dose and determination of LD_50_ value

Standardization of EMS (Ethyl Methane Sulfonate) mutagenesis treatment was done to determine LD_50_ value (lethal dose causing 50% reduction in seed germination) for the sunflower variety BBS-1 (BenchBio Sunflower-1). Kill curve analysis was done by treating 100 seeds with different EMS concentration (from 0 to 3% in deionized water) during 8 hours. Seeds were then thoroughly washed and transferred to wet tissue papers and kept in a growth chamber at 22°C with 12 hours photoperiod. This analysis was done in triplicate.

Finally, the optimal condition (0.6% EMS for 8 hours) was selected to mutagenise 25,000 M1 seeds with EMS. The seeds were sown in BenchBio Field 1–3, with a spacing of 60 cm × 45 cm. Standard cultivation practices were followed to grow the M1 plants. Self pollination was encouraged by covering inflorescence with a cloth bag before anthesis. Mature seed head were carefully tagged and collected from individual plants. The M2 seeds were harvested, packed and stored in cold room (8°C with 45-50% RH).

### Genomic DNA extraction

Ten seeds per M2 families were sown in small pots inside the glasshouse and leaf material was collected from 3rd and 4th leaf of eight individual plants per M2 family, 3 weeks post germination, in 96-well plates containing 2 steel beads (4 mm) per well. Tissues were ground using a custom built bead mill [38]. Approximately 100 mg was used for genomic DNA extraction using the Dneasy 96 Plant Kit (Qiagen, Hilden, Germany). The extracted genomic DNAs were run on a 1% agarose gel (HiMedia, India) to check quality. The quantity and purity of the genomic DNA samples were confirmed by UV spectrophotometer 1770 (Shimadzu Co., Japan). Individual DNA concentrations were normalized to 40 ng/ul and then used to prepare 8 fold pooling plates for TILLING screens.

### PCR amplification and mutation detection

PCR amplification is based on nested-PCR. The first PCR amplification is a standard PCR reaction using target-specific primers (Table 4) and 4 ng of sunflower genomic DNA. One μl of the first PCR served as a template for the second nested PCR amplification, using combination of specific primers labelled at the 5′end and 3′end with infra-red dyes IRD700 and IRD800 (Table 4), respectively. Mutation detection was carried out as described previously [31]. The identity of the mutations was determined by sequencing. TILLING request should be addressed to the corresponding author.

**Table 4:** Primers used

**Primers**	**Gene name**	**Sequence (5′..3′)**
BBPL0078F	FATA	CACTATACATAACCCACTCCGTACC
BBPL0136R	FATA	TTAACAAAAACCTAACCTGCAACAG
BBPL0138F	FATA	TGATGCATTTAGATGATGAATTTTG
BBPL0078R	FATA	AAAAGAAAGAACCGTGTTATCAGAG
BBPL0080F	FATA	TGAAATTTACAGATATCCTGCTTGG
BBPL0080R	FATA	GGATAAAAATGGCAAGATTTCAAAC
BBPL0082F	FATA	AAAGACTGAATTGCCCAAGTTTAC
BBPL0082R	FATA	CTATATCCCAGAATAGGCGAAATG
BBPL0146F_IRD700	FATA	CAAAACCTTCACACCTAACCTTTTC
BBPL0146R_IRD800	FATA	GTCTTATTAATCCCGACCTCATAAC
BBPL0147F_IRD700	FATA	CGTATTATAGGAGGTAGGAGGAAATC
BBPL0147R_IRD800	FATA	ATATCGTCATTGACTTTCTGGAGTC
BBPL0107F_IRD700	FATA	ATCAAAGATCATTCCAATGGTGAG
BBPL0107R_IRD800	FATA	ACGAATGGCATTTTACTCTTCTTG
BBPL0090F_IRD700	FATA	AAATTCGATGGAGTTAACGAGCTG
BBPL0090R_IRD800	FATA	AAGGATAAATACGAAACACAGATGC
BBPL0039F	SAD	AGACGTTGTTGACGTGAAACAC
BBPL0039R	SAD	CCATGGAAAGCAACAGAACC
BBPL0069F_IRD700	SAD	TTTGGGCGACTTATTTAATGC
BBPL0069R_IRD800	SAD	CTTGTTTTTTCAGAGCTTCAC

### Forward Genetic Screen (FGS)

Five seeds per M2 family were grown to maturity and phenotyped, at 4 months post-germination, in the field for traits related to yield and plant architecture. From each M2 plant, M3 seeds were collected and packed. Number of fertile M2 plants was also evaluated.

## Competing interests

The authors declare that they have no competing interests.

## Authors’ contributions

PKAK initiated EMS mutagenesis & standardization experiments, TILLING & maintaining TILLING population (MTP); ABo coordinated experiments, initiated forward genetic screens, participated in the bioinformatic analysis and carefully edited the manuscript; ABh drafted the MS, and participated in the mutant collection phenotyping; SP, ND carried the TILLING and bioinformatics analysis; AZ and AL provided critical inputs on genes to be TILLed; MC, AB conceived the study, coordinated experiments and carefully edited the manuscript. All authors read and approved the final manuscript.
